# Sports-related fractures in the geriatric population at a level I trauma center

**DOI:** 10.1186/s12877-024-05095-x

**Published:** 2024-05-27

**Authors:** Young Dae Jeon, Ki-Bong Park, Sang-Hun Ko, Jae-Min Oh, Sang-Gon Kim

**Affiliations:** Department of Orthopedic Surgery and Sports Medical Center, Ulsan University Hospital, University of Ulsan College of Medicine, 25 Daehakbyeongwon-ro, Dong-gu, Ulsan, 440333 Republic of Korea

**Keywords:** General sports trauma, Geriatric, Fracture, Epidemiology

## Abstract

**Background:**

The population is rapidly aging and remains active over the age of 65 years. An increasing number of sports-related fractures (SRFs) in individuals 65 and older are thus anticipated. Despite the increase in SRFs among the geriatric population, there are limited studies regarding the epidemiological data regarding SRFs in geriatric patients. This study examined the epidemiology of SRFs in a geriatric population who visited a level I trauma center.

**Methods:**

Data from geriatric patients who visited a level I trauma center were collected between June 2020 and July 2023. Overall, 1,109 geriatric patients with fractures were included in the study. Among them, 144 (13.0%) had fractures during sports activities (SRF group) and 965 (87.0%) had fractures during non-sports activities (non-SRF group). We investigated the type of sport in the SRFs and compared SRFs and NSRFs to describe the differences in patient, fracture, and treatment characteristics.

**Results:**

The mean age of SRFs was significantly lower (73.6 vs. 78.7 years; *P* < .001). The proportion of men was significantly higher in the SRF group than in the non-SRF group (51.4 vs. 29.6%; *P* < .001). We identified 13 types of sports associated with fractures, and the four most common were outdoor walking (36.1%), outdoor biking (27.8%), mountain hiking (19.4%), and gym (8.3%). There were no significant differences in the rate of hospitalization, operative treatment, or length of hospital stay between the two groups. However, compared to the non-SRF group, patients in the SRF group tended to return home after hospitalization (*P* = .002).

**Conclusion:**

This epidemiological study describes geriatric population that continues to be involved in sports and is thus susceptible to fractures. The identification of the type and distribution of SRFs in geriatric patients provides useful information for determining risk factors and appropriate preventive measures that may reduce their incidence.

## Background

The population is rapidly aging and remains more active over the age of 65 years [1]. Improvements in the standard of living and quality of health care combined with knowledge about the importance of exercise in maintaining health have led to an increased number of geriatric patients participating in sporting activities [2]. All forms of exercise, from walking and gardening to swimming, tennis, and biking, are known to improve health and well-being [1]. Recent publication found that biking, swimming, mountain hiking, walking/jogging, and alpine skiing are the most popular sports among older adults [3, 4].

It is expected that there will be an increase in sports-related injuries, especially fractures, in this age group [56]. However, there is insufficient information on the incidence of sports-related fractures (SRFs) and which exercises can be safely recommended for the geriatric population. Epidemiological data regarding SRFs are mostly limited to children and adolescents [5–9] and to the author’s knowledge, there was no study regarding the SRFs in the geriatric population. Park et al. [10] studied overall sports-related injuries, including not only fractures but also dislocations, contusions, sprains, and wounds, but only studied sports-related injuries that occurred in the extremities, excluding head, neck, or trunk injuries. Imam et al. [11] studied the elderly population aged 65 years or older and analyzed comprehensive sports-related injuries that were not limited to fractures.

Therefore, this study aimed to analyze the epidemiology of SRFs in a geriatric population. Second, SRF characteristics were compared with those of other non-SRFs (NSRFs) in a clinical setting, to understand and describe the differences to support clinical decision-making regarding treatment management. We hypothesized that the epidemiology of SRFs in geriatric population would differ age and sex and would experience more favorable discharge dispositions.

## Methods

### Ethics

This retrospective study was conducted with the approval of the Institutional Review Board (IRB) of our institution (IRB No. 2023-10-001). The requirement for patient consent was waived because of the retrospective nature of the study.

### Participants and inclusion criteria

**Fig. 1:**
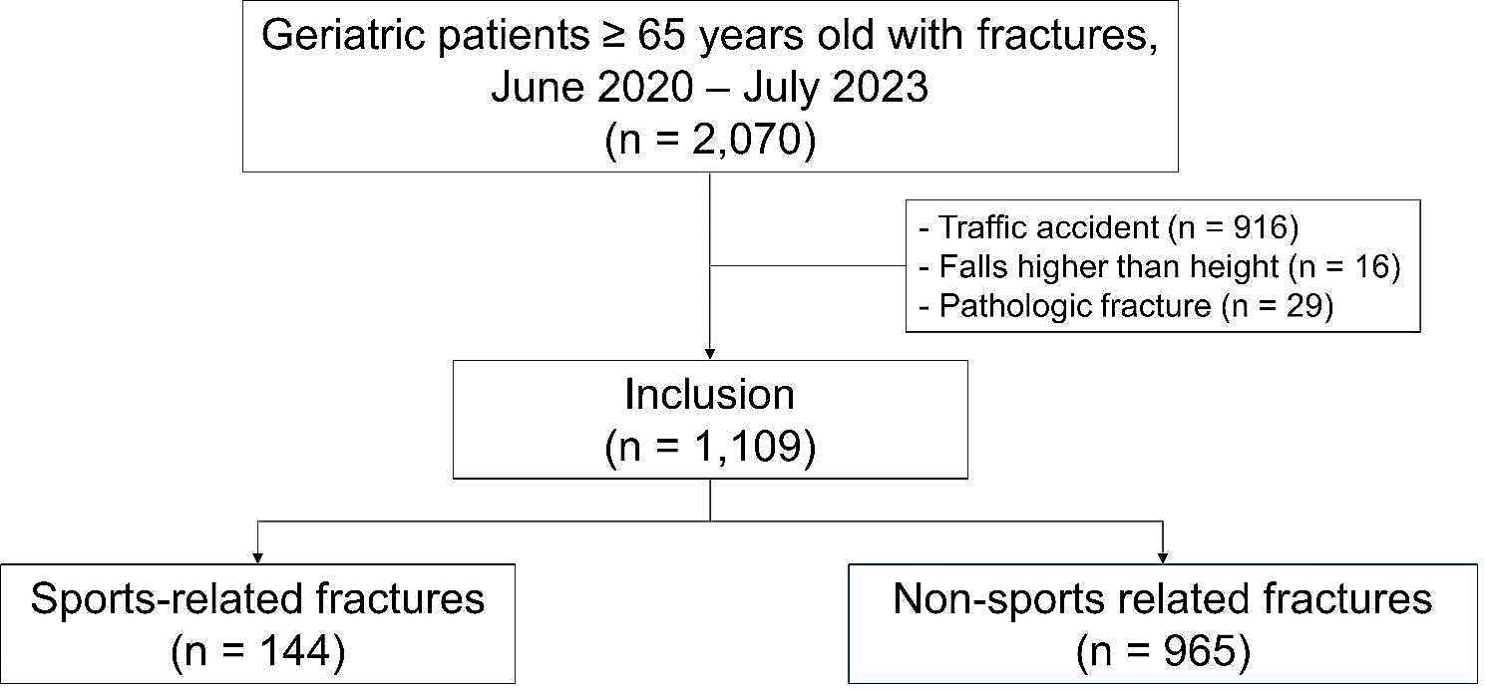
Flow-chart

Adults 65 years and older sustaining an acute fracture were included in this study. From June 2020 to July 2023, a total of 2,070 patients were treated at our level one trauma center (Fig. 1).

Medical and radiological records were assessed by two orthopedic surgeons. Patients with fractures after traffic accidents (*n* = 916), fractures after falling from a height (*n* = 16), and pathological fractures (*n* = 29) were excluded. Overall, 1,109 patients were included in this study. The definition of sport is difficult, particularly in the elderly, where walking may reasonably be defined as a sport [12]. We defined sport as a type of activity for improving physical activity and classified simple walking to move from one place to another as non-sports. Among 1,109 patients, 144 (13.0%) had fractures during sports activities (SRF group) and 965 (87.0%) had fractures during non-sports activities (NSRF group) [13].

### Evaluation

We documented the type of sport played at the time of injury, age, sex, season in which the fracture occurred, location of injury, anatomical region of the fracture, hospitalization (yes or no), hospital length of stay (LOS), and treatment method (operative or conservative). Patients were separated into groups by age (65–69, 70–74, 75–79, 80–84, 85–89, 90–94, ≥ 95 years). The seasons in which fractures occurred were divided into spring (March, April, and May), summer (June, July, and August), autumn (September, October, and November), and winter (December, January, and February). We classified fractures according to the site of the fracture (head, chest, back, upper extremities, and lower extremities), based on the previous study.^5^ We compared SRFs and NSRFs to describe the differences in patient, fracture, and treatment characteristics and evaluated the possible risk factors.

### Statistical analysis

Continuous variables are presented as mean with standard deviation. Normal distribution of data was checked by descriptive measures such as coefficients of skewness and kurtosis. Continuous variables were compared with Student’s t-test, and categorical variables were compared with the Chi-square test or Fisher’s exact test, as appropriate. The risk of SRF according to underlying disease was analyzed using logistic regression. All analyses were performed in IBM SPSS Statistics for window, version 28 (IBM Corp., Armonk, NY). Statistical significance was set *P* < .05.

## Results

**Fig. 2:**
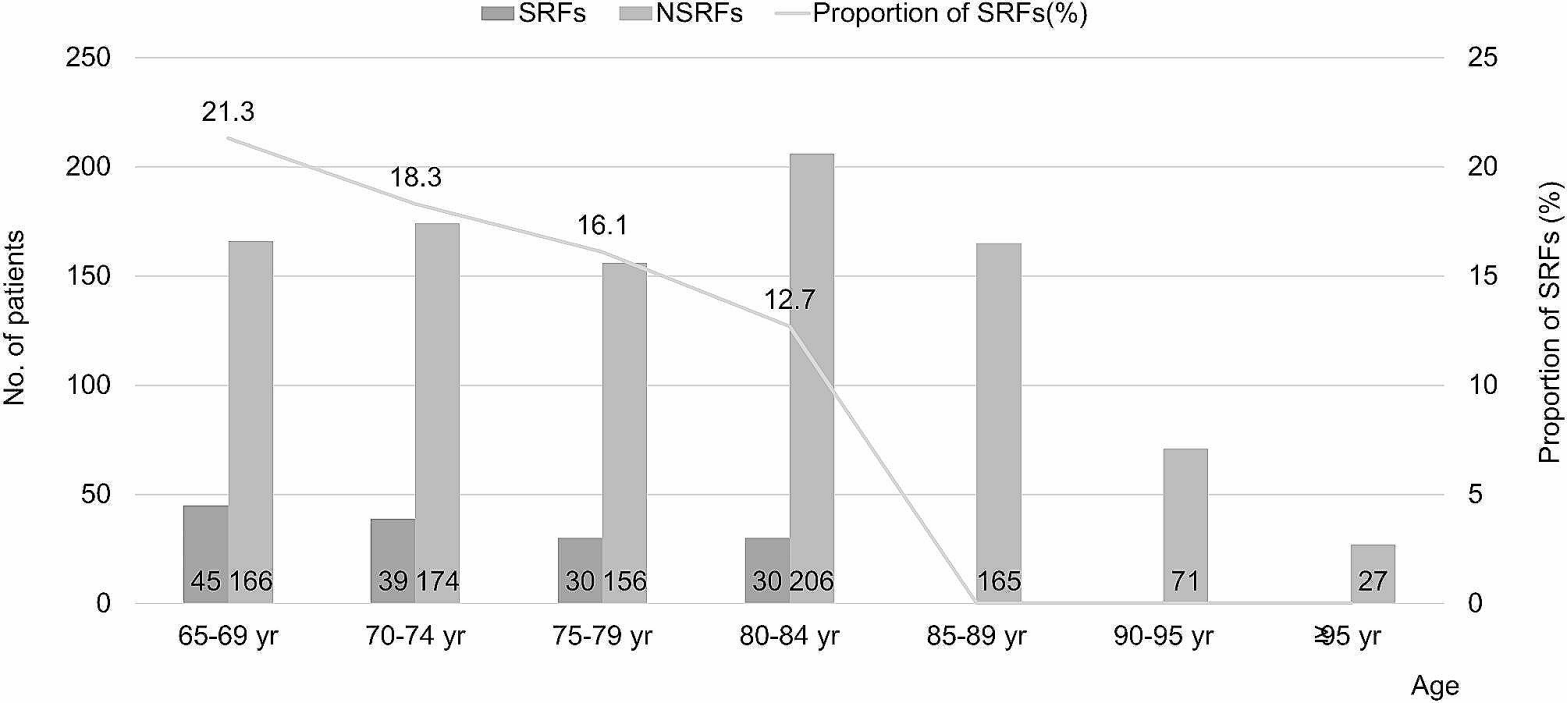
The incidence of sports-related and non-sports related geriatric fractures by age distribution. SRFs: sports-related fractures, NSRFs: non-sports related fractures

The basic demographics of the two groups are presented in Table 1. The mean age of geriatric patients with SRFs was 73.6 ± 6.1 years, which was significantly different from that of geriatric patients with NSRFs (78.7 ± 8.4 years; *P* < .001). The numbers of SRFs and NSRFs according to age distribution are shown in Fig. 2. SRFs occurred most frequently in the 65–69 year age group (31.3%), followed by the 70–74 year age group (27.1%). As patient age increased, the number of SRFs decreased, and there were no SRFs in patients aged > 85 years. In the NSRF group, the 80–84 year age group accounted for 21.3%, followed by the 70–74 year age group (18.0%). The rate of SRFs based on the overall incidence per age group is also shown.

**Table 1:** Demographics of sports-related and non-sports-related fractures in the geriatric population

	All Participants (*N* = 1,109)	Sports-related Fractures Group (*N* = 144)	Non-sports-related Fractures Group (*N* = 965)	*P*-value
Sex, male/female	360 (32.5)/749 (67.5)	74 (51.4)/70 (48.6)	286 (29.6)/679 (70.4)	0.000
Age, y	78.0 ± 8.3	73.6 ± 6.1	78.7 ± 8.4	0.000
Season				0.021
Spring	263 (23.7)	27 (18.8)	236 (24.5)	
Summer	331 (29.8)	41 (28.5)	290 (30.1)	
Autumn	280 (25.2)	51 (35.4)	229 (23.7)	
Winter	235 (21.2)	25 (17.4)	210 (21.8)	
Outdoor/indoor				0.000
Outdoor	398 (35.9)	126 (87.5)	272 (28.2)	
Indoor	711 (64.1)	18 (12.5)	693 (71.8)	
Comorbidity				
Diabetes	313 (28.5)	45 (31.5)	268 (28.0)	0.396
Hypertension	551 (50.1)	67 (46.9)	484 (50.6)	0.400
CVA	196 (17.9)	7 (4.9)	189 (19.8)	0.000
Parkinson	24 (2.2)	1 (0.7)	23 (2.4)	0.351
Heart	150 (13.7)	18 (12.6)	132 (13.8)	0.685
Kidney	84 (7.7)	8 (5.6)	76 (8.0)	0.318
Osteoporosis	83 (7.6)	7 (4.9)	76 (7.9)	0.197
Osteopenia	6 (0.5)	1 (0.7)	5 (0.5)	0.568

Data are presented as mean ± standard deviation or n (%). CVA: cerebrovascular accident

In the SRF group, of 144 patients, 70 (48.6%) were women and 74 (51.4%) were men. In contrast, in the NSRF group of 965 patients, the percentage of women (70.4%; *n* = 679) was much higher than that of men (29.6%; *n* = 286). The proportion of men was significantly higher in the SRF group than in the NSRF group (*P* < .001).

SRFs were most prevalent in the seasonal order of autumn (35.4%), summer (28.5%), spring (18.8%), and winter (17.4%), and NSRFs were most prevalent in the seasonal order of summer (30.1%), spring (24.5%), autumn (23.7%), and winter (21.8%). There was a significant difference in the seasonal distribution of geriatric fractures in the two groups (*P* = .021).

In the SRF group, the number of outdoor and indoor fractures were 126 (87.5%) and 18 (12.5%), respectively. In the NSRF group, indoor fractures accounted for 71.8% of all fractures, higher than the number of outdoor fractures (28.2%). The rate of outdoor fractures was 87.5% in the SRF group, significantly higher than the 28.2% in the NSRF group (*P* < .001).

Overall, 13 sports were associated with SRFs in the geriatric population (Table 2). Eight sports activities (61.5%) were outdoor activities, and the remaining five sports activities (38.5%) were indoor activities. The four dominant sports associated with SRFs were outdoor walking (52 patients; 36.1%), outdoor biking (40 patients; 27.8%), mountain hiking (28 patients; 19.4%), and gym (12 patients; 8.3%). Table 3 shows the characteristics of the four most prevalent sport types in the SRF group. The sex distribution in gyms, outdoor walking exercises, and mountain hiking was female-dominated (83.3%, 67.3%, and 60.7%, respectively), and in outdoor biking, it was male-dominated (92.5%). During outdoor walking, mountain hiking, and outdoors biking, lower extremity fractures were developed most commonly (40.4%, 35.8%, and 31.8%, respectively). During gym, back fractures were the most common at 33.3%. The proportion of SRFs requiring surgical treatment was highest in the following order: mountain hiking (62.1%), outdoor biking (52.4%), outdoor walking (44.4%), and gym (41.7%).

**Table 2:** Sports involved in Sports-related Fractures in the Geriatric Population

Outdoor/indoor	Sports	*N* (%)
Outdoor	Outdoor walking	52 (36.1%)
	Outdoor biking	40 (27.8%)
	Mountain hiking	28 (19.4%)
	Golf	2 (1.4%)
	Tennis	1 (0.7%)
	Outdoor swimming	1 (0.7%)
	Soccer	1 (0.7%)
	Paragliding	1 (0.7%)
Indoor	Gym	12 (8.3%)
	Indoor walking	2 (1.4%)
	Indoor biking	2 (1.4%)
	Table tennis	1 (0.7%)
	Badminton	1 (0.7%)

**Table 3:** Characteristics of the Four Most Prevalent Sports Types in the Sports-related Fracture Group

	Outdoors walking	Outdoors biking	Mountain hiking	Gym
No. of patients	52	40	28	12
No. of fractures	52	44^*^	31^*^	12
Age, y	74.1 ± 5.9	73.6 ± 6.6	71.9 ± 4.8	74.2 ± 6.8
Sex				
Female	35 (67.3%)	3 (7.5%)	17 (60.7%)	10 (83.3%)
Male	17 (32.7%)	37 (92.5%)	11 (39.3%)	2 (16.7%)
Region of fracture				
Lower extremities	21 (40.4%)	14 (31.8%)	11 (35.5%)	3 (25.0%)
Upper extremities	18 (34.6%)	13 (29.5%)	10 (32.3%)	2 (16.7%)
Face	7 (13.5%)	9 (20.5%)	2 (6.5%)	3 (25.0%)
Back	3 (5.8%)	4 (9.1%)	6 (19.4%)	4 (33.3%)
Head	2 (3.8%)	3 (6.8%)	1 (3.2%)	-
Chest	1 (1.9%)	1 (2.3%)	1 (3.2%)	-
Operative treatment	24 (46.2%)	21 (52.5%)	17 (60.7%)	5 (41.7%)

Data are presented as mean or *n* (%)

^*^ Includes multiple fractures

Table 4 shows the fracture characteristics of the two groups. The proportion of single fractures was 95.1% and 96.6% in the SRF and NSRF groups, respectively. The number of patients with simultaneous fractures in two or more areas was 7 (4.9%) and 33 (3.4%) in the two groups, respectively, with no statistically significant difference (*P* = .387). The presence or absence of SRFs was related to the region of fracture (*P* < .001). In the SRF group, more fractures were observed in the upper extremities, face, head, and chest than expected, and in the NSRF group, more lower extremities and back fractures were observed than expected.

**Table 4:** Characteristics of Sports-related and Non-sports-related Fractures

	Sports-related Fractures	Non-sports-related Fractures	*P* value
Number of fractures			
Single	137 (95.1)	932 (96.6)	0.387
Multiple	7 (4.9)	33 (3.4)	
Total	144	965	
Region of fracture			< 0.001
Lower extremities	53 (35.1)	495 (49.5)	
Upper extremities	47 (31.1)	241 (24.1)	
Face	22 (14.6)	64 (6.4)	
Back	18 (11.9)	153 (15.3)	
Head	7 (4.6)	23 (2.3)	
Chest	4 (2.6)	23 (2.3)	
Total	151^*^	999^*^	

^*^ Includes multiple fractures

Table 5 shows the treatment characteristics of the two groups. The hospitalization rates in the SRF and NSRF groups were 63.9% and 67.4%, respectively, with no significant difference (*P* = .409). The proportion of patients who received operative treatment after hospitalization in the SRF and NSRF groups were 50.7% and 55.0%, respectively (*P* = .331). In entire population, hospital LOS in the SRF and NSRF groups was 14.3 and 14.5 days, respectively, with no statistically significant difference (*P* = .894). However, hospital LOS for patients with spinal fractures in the SRF group (29.0 days) was significantly longer than that in the NSRF group (11.6 days, *P* < .001). In entire population, the proportion of patients who were able to return home immediately after hospitalization was 70.7% in the SRF group, which was significantly higher than that in the NSRF group (54.2%; *P* = .002). In a sub-analysis according to region of fracture, 73.7% of lower extremities fracture patients in the SRF group were discharged home after hospitalization, and this rate was significantly higher than that of lower extremities fracture patients in the NSRF group (45.5%, *P* < .001).

**Table 5:** Characteristics related to the Management of Sports-related and Non-sports-related Fractures

	Sports-related Fractures	Non-sports-related Fractures	*P* value
Hospitalization	92 (63.9%)	650 (67.4%)	0.409
Operative treatment	73 (50.7%)	531 (55.0%)	0.331
Hospital length of stay			
Overall	14.3 (1–64)	14.5 (1–168)	0.894
Lower extremities	14.6 (4–64)	17.4 (1–168)	0.261
Upper extremities	8.0 (1–26)	8.5 (1–63)	0.793
Face	8.0 (7–10)	8.8 (1–23)	0.835
Back	29.0 (3–64)	11.6 (1–96)	< 0.001
Head	11.0 (3–31)	7.9 (1–33)	0.390
Chest	5.0 (4–7)	18.7 (1–51)	0.447
Discharge home			
Overall	65 (70.7%)	352 (54.2%)	0.002
Lower extremities	28 (73.7%)	185 (45.5%)	< 0.001
Upper extremities	21 (87.5%)	90 (73.2%)	0.137
Face	3 (75.0%)	9 (81.8%)	0.789
Back	5 (41.7%)	55 (64.0%)	0.141
Head	5 (71.4%)	10 (45.5%)	0.246
Chest	3 (100%)	3 (100%)	-

Data are presented as mean (range) or n (%)

Table 6 shows the risk factors for SRF due to the patient’s underlying disease, and the risk of SRF was reduced by 0.2 times when cerebrovascular accident (CVA) was present compared to without CVA, confirming statistical significance (*P* < .001).

**Table 6:** Risk Factor for Sports-related Fractures according Patient’s Underlying Disease

	Odds Ratio	95% C.I	*P*-value
Diabetes	1.179	0.806–1.724	0.936
Hypertension	0.860	0.605–1.223	0.400
CVA	0.208	0.096–0.453	0.000
Parkinson diseases	0.286	0.038–2.134	0.222
Heart conditions	0.897	0.529–1.519	0.685
Kidney diseases	0.684	0.323–1.449	0.321
Osteoporosis	0.596	0.269–1.320	0.202
Osteopenia	1.339	0.155– 11.548	0.790

C.I: Confidence Interval, CVA: cerebrovascular accident

## Discussion

The most important findings of the present study were SRFs occurred at a relatively younger age than NSRFs and showed a close male-to-female ratio compared with female-dominant NSRFs. The four most prevalent sports activities that caused SRFs were outdoor walking, outdoor biking, mountain hiking, and gym. When comparing SRFs and NSRFs, geriatric patients with SRFs tended to return home after hospitalization; however, the rates of hospitalization, operative treatment, or hospital LOS did not differ between the groups.

This study showed that 13.0% of geriatric patients with fractures were injured during sports activities. Court-Brown et al. [12] studied the epidemiology of acute SRFs in adults and reported that 12.8% of all fractures were SRFs. Wood et al. [9] reported that 23.9% of adolescent fractures were caused by sports activities. The incidence of SRFs in the geriatric population was similar to the incidence in the general adult population, but was lower than the incidence in the adolescent population.

In this study, in geriatric patients over 65 years of age, SRFs tended to gradually decrease with age. This is similar to the results of other studies in which the proportion of minor injuries and wounds gradually increased with age, whereas the proportion of SRFs gradually decreased [14]. We found that the proportion of SRFs among all geriatric fractures was higher in men (74/360, 20.6%) than in women (70/749, 9.3%). Osteoporotic fractures in geriatric patients are known to occur more commonly in women and the lifetime risk of any osteoporotic fracture lies within the range of 40–50% in women and 13–22% in men [15, 16]. However, for SRFs, the incidence in males and females was reported to be 261/10^5^ and 35/10^5^, respectively [12]. Previous studies reported that the comparatively higher number of sports injuries in males may be explained by the higher percentages of men participating in sports [9, 10, 14, 17].

In this study, SRFs occurred in the following order of prevalence: lower extremities, upper extremities, face, back, head, and chest. Although the classification criteria were different from those used in the present study, in a European study, SRFs occurred in the following order of prevalence: skull (26.1%), shoulder and upper arm (17.0%), rib and thoracic spine (14.8%), forearm and wrist (14.6%), lower leg and ankle (13.5%), femur (7.5%), and lumbar spine and pelvis (6.5%) [14]. SRFs in this study mainly occurred in sports with a low level of activity, such as outside walking, whereas SRFs in the European study occurred in high-activity sports, such as alpine skiing. Therefore, for this reason, it can be inferred that differences may occur in the region of prevalent fractures in the two studies.

A recent European publication found cycling, swimming, mountain climbing, walking/jogging, and alpine skiing to be the most popular sports among older adults [14]. A recent Japanese publication revealed that the top three most popular types of sports among geriatric men were golf, walking, and ground golf, while the top three most popular types of sports among geriatric women were fitness exercise, walking, and weight training [18]. We identified 13 types of sports associated with SRFs in the geriatric population. A study on geriatric sports-related injuries conducted at a level one trauma center located in a mountainous tourist region for winter sports reported the frequency of fracture by sport, and the results showed that alpine skiing (41.0%) was the most common, followed by cycling (24.7%) and mountain climbing (16.8%) [14]. In this study, the three sports that were most associated with SRFs in the geriatric population were outdoor walking, outdoor biking, and mountain hiking, which are all outdoor activities. Sports that cause SRFs in adolescents and adults include high-level activities such as football, rugby, skiing, snowboarding, hockey, and basketball [9, 10]. Because there are differences between the sports that the elderly population frequently participates in and the sports in which SRFs frequently occur, additional research on the incidence of SRFs by sports type is needed. Additionally, unlike the youth or adult population, information on low-level activities should be collected first.

This study showed that outdoor biking accounted for 27.8% of all SRFs and ranked second among all the causes of SRFs. It has been reported that among sports-related injuries occurring in the geriatric population aged 65 years or older, fractures during bicycle use were the most frequent diagnosis [1]. In Sweden, approximately half of fatally injured bicyclists are 65 years or older and the annual injury incidence in this age group was 2.4 and 2.2 per 1,000 men and women, respectively [19]. A study assessing the national incidence and trends of bicycle injuries found that injuries among elderly patients are becoming more common, with a high rate of fracture and head injury [20]. A previous study on bicycle-related injuries reported that among lower extremity injuries (88 cases), fractures of the tibia/fibula, ankle, or foot accounted for 30.6% [21].

This study showed that mountain hiking accounted for 19.4% of all SRFs and ranked third among all the causes of SRFs. Mountain hiking injuries were more common among people aged 65 years and older (38.0%) than among those aged 45–64 years (23.6%) [11]. Irregular and unstable terrain found on mountains can cause fractures of the hand, ankle, and wrist and ligament injuries of the ankle [14, 22]. In this study, the rate of operative treatment for SRFs was highest in the mountain hiking group (62.1%).

This study showed that the rate of home discharge after hospitalization in the SRF group was significantly higher than that in the NSRF group. Although the level of activity before fracture was not directly compared, it can be reasonably assumed that the activity level in the SRF group participating in sports activities was higher than that in the NSRF group. These results are similar to those of previous studies, which reported that poor premorbid mobility or sarcopenia is a predictor for non-home discharge [23, 24].

Although the patient’s underlying disease did not increase the risk of developing SRF, CVA lowered the risk of developing SRF compared to patients without CVA. The authors reasoned that CVA may have resulted in a decrease in the occurrence of SRF because patients with a history of CVA gradually decreased muscle strength after the onset of CVA, which naturally limited participation to sport activities [25].

This study provides useful information by using data from patients in a clinical setting. However, this study has certain limitations. First, our data were collected from a single trauma center, which may have inhibited its applicability to other geographic settings. Second, we could not evaluate sports-related soft tissue injuries, such as sprains, ligament injuries, and muscle and tendon injuries. Third, a major limitation of this study was its retrospective design. Factors such as individual skill levels and weather that affect sports-related injuries could not be reflected. Fourth, the magnitude of injuries was not identified. This study aimed to develop strategies to prevent SRFs in geriatric populations. We examined patients who had already been injured. Therefore, we could not identify community population- or participant-based incidences. Finally, it is possible that the severity of injuries may have been overestimated because of measurements during trauma center visits. Injured patients often seek medical care at local outpatient clinics when their pain is not severe, and if there is no motion restriction, the number of patients with fractures presenting to the trauma center may become skewed.

## Conclusions

This epidemiological study describes a growing geriatric population that continues to be involved in sports and is thus susceptible to fractures. Identification of the type, mechanism, and distribution of sports-related geriatric fractures provides useful information for determining risk factors and appropriate preventive measures that may reduce their incidence.

## Data Availability

The datasets used and/or analyzed during the current study are available from the corresponding author on reasonable request.

## Abbreviations

SRF: sports-related fracture
NSRF: non-sports-related fracture
LOS: length of stay
CVA: cerebrovascular accident
